# Lepromatous leprosy infection from *Mycobacterium lepromatosis* without known risk factors or exposures in the Upper Midwest United States

**DOI:** 10.1016/j.jdcr.2023.12.015

**Published:** 2024-01-07

**Authors:** Hannah B. Fleischmann, Nandhini Sureshkumar, Erik J. Stratman

**Affiliations:** a University of Wisconsin School of Medicine and Public Health, Madison, Wisconsin; b Department of Internal Medicine, Marshfield Clinic Health System, Marshfield, Wisconsin; c Department of Dermatology, Marshfield Clinic Health System, Marshfield, Wisconsin

**Keywords:** erythema nodosum leprosum, leprosy, *Mycobacterium lepromatosis*, skin infection, Wisconsin

## Introduction

Leprosy is an infectious disease primarily affecting skin, peripheral nerves, upper respiratory tract, and eyes. Untreated, the disease can lead to significant physical and neurologic impairments. Leprosy was initially thought to be caused exclusively by *Mycobacterium leprae* infection, but *Mycobacterium lepromatosis* was identified as a cause of human leprosy in 2008.^1^ Since its discovery, at least 22 reports of human *M lepromatosis* infection have been reported.^2^

*M lepromatosis* infection demonstrates variable disease manifestations, including diffuse lepromatous leprosy and inflammatory reactions, as well as clinical overlap with other diseases.^3^, ^4^, ^5^, ^6^, ^7^ Although *M leprae* infections have demonstrated zoonotic spread through handling and consumption of nine-banded armadillos, zoonotic spread of *M lepromatosis* to humans has not been proven, although it has been detected in small subsets of red squirrels in England, Ireland, and Scotland.^8^ United States patients with *M lepromatosis* typically are immigrants or travelers from endemic regions and are diagnosed most often in urban tropical medicine clinics. *M lepromatosis* remains a relatively novel organism in the rural Midwest United States, with much to be learned about its spread, diagnosis, treatment, and prevention.

This case report highlights an unusual presentation of *M lepromatosis* in a patient without known risk factors or exposures, raising new questions regarding the spread of *M lepromatosis* and encouraging dermatologist awareness regarding this potentially emerging insidious infection.

## Case report

A 77-year-old man was referred to dermatology for evaluation of skin lesions 2 weeks following an acute care visit for presumed lower extremity cellulitis treated with oral cephalexin. The patient was experiencing persistent right leg tenderness and malaise. Past history was notable for progressive bilateral ulnar and median nerve neuropathy for months and seropositive rheumatoid arthritis for 13 years, controlled with hydroxychloroquine, methotrexate, and prednisone.

**Fig 1:**
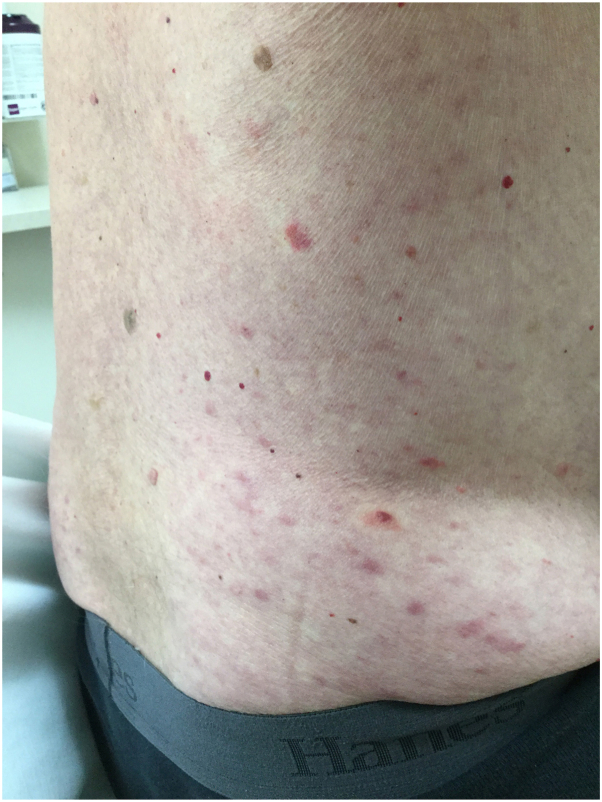
Incidental inflammatory trunk papules in patient with progressive neuropathy, rheumatoid-like arthralgias and leg soft tissue inflammation consistent with a type 2 reaction.

**Fig 2:**
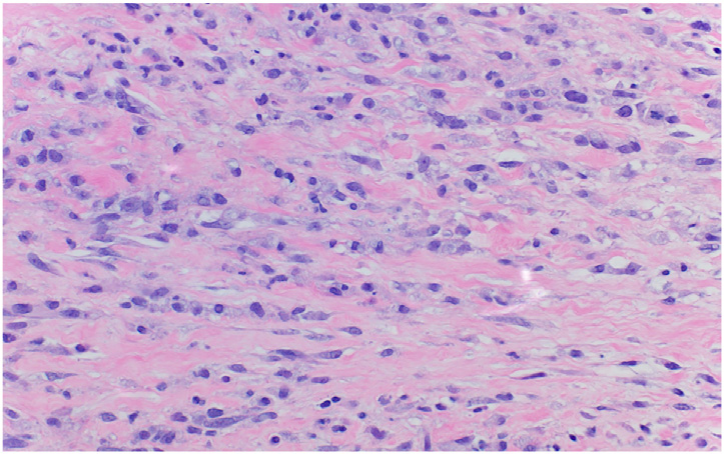
Dermal interstitial lymphohistiocytic and granulomatous inflammation (Hematoxylin-eosin stain; original magnification: ×40).

**Fig 3:**
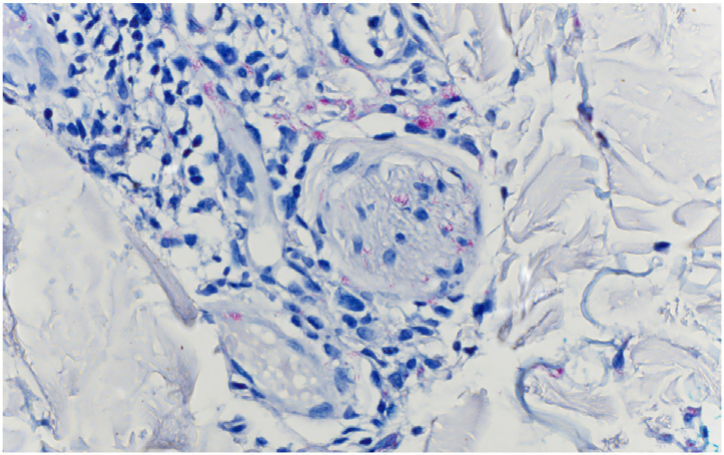
Fite stain revealing multiple intraneural acid-fast bacilli in areas of granulomatous inflammation (Fite stain; original magnification: ×40).

On examination, the lower portion of the right leg demonstrated scattered inflamed, tender nodules. The left medial calf had a well-demarcated indurated inflammatory plaque with desquamation. Unbeknownst to the patient, there were also several discrete, faintly erythematous, ill-defined macules, and papules scattered over flanks and abdomen (Fig 1). Hematoxylin and eosin, Brown-Hopps, Fite, and Gomori Methenamine Silver stains were obtained from lower extremity lesions. Results demonstrated lymphohistiocytic and granulomatous inflammation with areas of neutrophils and staining for acid-fast bacilli, suggesting a mycobacterial infection with type 2 reaction (Figs 2 and 3). Tissue polymerase chain reaction testing was negative for tuberculosis and mycobacterium avium complex.

**Fig 4:**
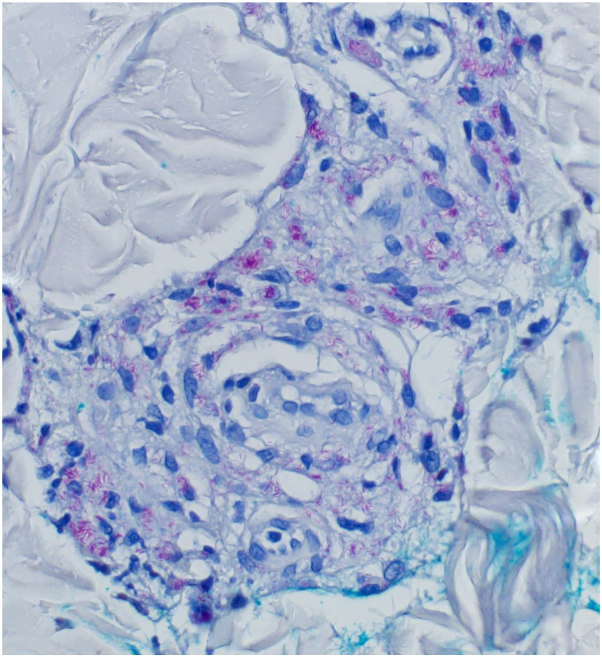
Large numbers of persistent mycobacteria in skin (Fite stain; original magnification: ×40).

When the patient returned for cultures, leg lesions were improving significantly after completion of 14 days of cephalexin, but violaceous papules on the back remained. The patient reported increasing fatigue, malaise, weight loss, progressive peripheral neuropathy, and flaring of rheumatoid arthritis symptoms. Additional skin biopsy was performed on the low back, also consistent with mycobacterial infection (Fig 4). Tissue cultures were negative and skin biopsies were sent for broad range bacterial polymerase chain reaction and sequencing, which revealed *M lepromatosis*.

The patient was diagnosed with lepromatous leprosy with type 2 reaction (erythema nodosum leprosum). The case was reported to Wisconsin Department of Health Services. Exposure history was reviewed, and no obvious risk factors were identified. The patient spent most of his life in Wisconsin working as a paper mill chemist, with brief periods of time in Arkansas and Mississippi 20 years prior. Travel history was notable for brief vacations to Ireland, Canada, Hawaii, and Alaska in the past decade, but none within recent years. He had no direct exposure to armadillos or red squirrels.

A 24-month treatment regimen of moxifloxacin, minocycline, and rifampin was initiated at the direction of the National Hansen’s Disease Program. Although the patient initially continued all antirheumatoid arthritis medications, his improvement on antimicrobial therapy led to arthritis medication tapering and nerve release surgery cancellation.

## Discussion

*M lepromatosis* is a relatively new implicated organism causing leprosy. Even though the symptoms and treatments of leprosy caused by *M lepromatosis* and *M leprae* are similar, more research on *M lepromatosis* is needed to determine additional risk factors for infection. This case highlights the challenges in the diagnosis of an unusual variant of leprosy in a patient with no identifiable risk factors.

Another diagnostic challenge highlighted here is the overlap between leprosy, its inflammatory reactions, and rheumatologic disease. Leprosy and its immunologic reactions can mimic rheumatologic diseases, including rheumatoid arthritis, systemic lupus erythematosus, spondylarthritis, sarcoidosis, relapsing polychondritis, and vasculitis.^5^, ^6^, ^7^ Patients with leprosy can also have positive rheumatoid factor, anticitrullinated peptide, antinuclear antibodies, and antineutrophil cytoplasmic antibodies.^5^^,^^6^ Diagnostic errors and delays in diagnosis can occur when these autoantibodies are found in leprosy patients presenting with rheumatologic manifestations. It is unclear whether our patient’s arthritis was truly rheumatoid arthritis or leprosy related. His arthritis symptoms improved with adding antimicrobial therapy, leading to chronic rheumatoid medication tapering. This suggests a possible leprosy role in his chronic arthritis. It is unknown if chronic arthritis in leprosy is due to intraarticular bacillus, such as infectious arthritis, an immune response producing circulating immune complexes resulting in joint inflammation, or a reaction to bacterial antigens, similar to reactive arthritis.^5^ Clinicians should consider leprosy in the differential diagnosis of rheumatoid arthritis, especially when patients fail to improve with initial therapies and have combinations of cutaneous, neurologic, and musculoskeletal symptoms. This is true even in geographic areas with low leprosy prevalence, including the United States.

The lack of relevant exposure history in this case suggests there may be specific risk factors for *M lepromatos*is transmission not yet identified. The geographic reach of this disease may be much broader than previously thought. This raises questions about the transmission of *M lepromatosis* in the United States. There may be silent reservoirs of infectivity with this emerging mycobacterial organism. Although *M leprae* has been identified in armadillo populations with human transmission documented, *M lepromatosis* has not been detected in the armadillo population nor in any endemic animal population in the United States. Environmental scanning to identify vectors in the United States should continue. Given the low prevalence of leprosy in the United States, clinical suspicion is often low or nonexistent. Further studies should investigate whether leprosy caused by *M lepromatosis* is more common in the developed world than previously thought. Clinicians should consider leprosy in patients presenting with cutaneous, rheumatologic, and neurologic sequelae.

## Conflicts of interest

None disclosed.
